# Purine limitation prevents the exogenous pyridoxal 5′-phosphate accumulation of *Salmonella enterica yggS* mutants

**DOI:** 10.1128/spectrum.02075-24

**Published:** 2024-10-22

**Authors:** Kailey S. Ezekiel, Diana M. Downs

**Affiliations:** 1 Department of Microbiology, University of Georgia, Athens, Georgia, USA; Emory University School of Medicine, Atlanta, Georgia, USA

**Keywords:** YggS, PLPHP, PLP homeostasis, purine limitation

## Abstract

YggS belongs to the highly conserved pyridoxal 5′-phosphate (PLP) binding protein family (COG0325) that is found in all domains of life. Though no precise biochemical activity or molecular mechanism has been determined for this protein, an involvement in vitamin B_6_ homeostasis has been demonstrated in multiple organisms. In *Salmonella enterica*, loss of YggS results in altered B_6_ vitamer pools, including an accumulation of PLP in the growth medium. Transposon mutagenesis identified an insertion upstream of *purC* (encoding 5′-phosphoribosyl-5-aminoimidazole-4-*N*-succinocarboxamide synthetase, EC 6.3.2.6) that eliminated accumulation of PLP in the spent medium. Genetic characterization of the insertion showed the causative effect was reduced expression of *purC*, which limited purine biosynthesis. Data herein shows that purine limitation decreased the exogenous accumulation of B_6_ vitamers of a *yggS* mutant but did not suppress other *yggS* mutant phenotypes. Neither limitation for ATP, regulation by PurR, or decreased growth rate, all of which are direct consequences of purine limitation, prevented exogenous B_6_ vitamer accumulation of a *yggS* mutant. This work establishes a relationship between the status of purine biosynthesis and the impact of a *yggS* mutation. It lays the foundation for continued efforts to identify the physiological role of YggS and its homologs.

**IMPORTANCE:**

Pyridoxal 5′-phosphate is the active form of vitamin B_6_ and is an essential cofactor in all domains of life. PLP can be synthesized *de novo* or salvaged from the environment from one of the six B_6_ vitamers. B_6_ vitamer levels appear to be tightly regulated, and alterations in their levels can have deleterious effects, most notably being the development of B6-dependent epilepsy in humans. YggS homologs are broadly conserved across multiple organisms and considered to be involved in maintaining B_6_ homeostasis, though no specific mechanism has been defined. The current study showed that the exogenous accumulation of PLP caused by a lack of YggS can be prevented by purine limitation. The demonstration that purine limitation impacts exogenous PLP accumulation separates one consequence of a *yggS* mutation for further study and contributes to continuing efforts to define the biochemical and physiological roles of the COG0325 family of proteins.

## INTRODUCTION

**Fig 1:**
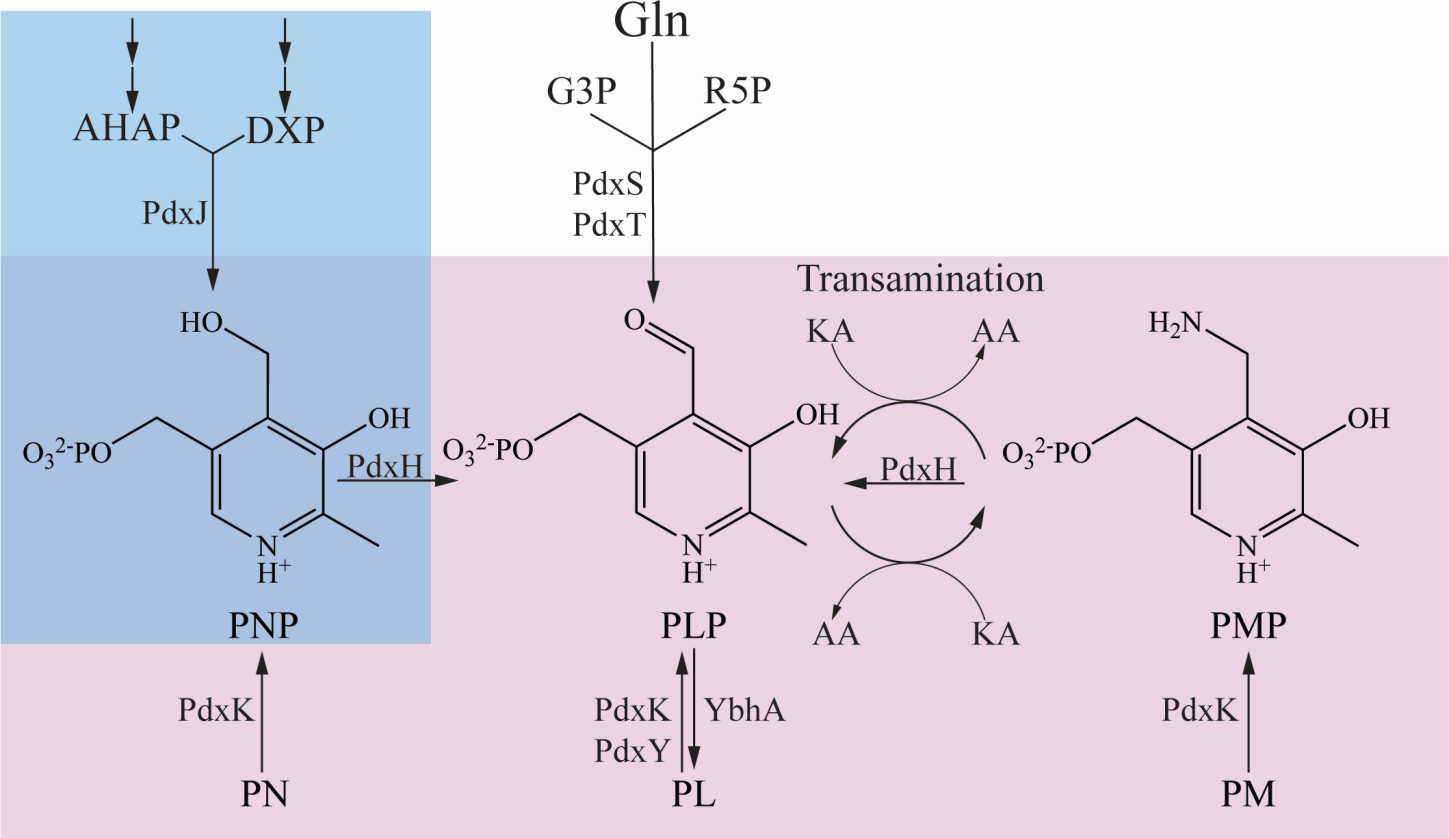
Pathways of vitamin B_6_ synthesis and salvage. Pathways that synthesize and/or salvage B6 vitamers are schematically shown. The blue box highlights steps unique to the DXP-dependent pathway, which is present in *S. enterica*. The product PNP is oxidized to PLP by PdxH. In the more common DXP-independent pathway, PLP is synthesized from Gln, G3P, and R5P by a single-enzyme complex (PdxST). B_6_ vitamers can also be salvaged (pink box) from the environment, phosphorylated by kinases, and ultimately converted to PLP. Gln, glutamine; G3P, glyceraldehyde 3-phosphate; R5P, ribose 5-phosphate DXP, deoxyxylulose 5-phosphate; AHAP, 3-amino-1-hydroxyacetone 1-phosphate; PL, pyridoxal; PN, pyridoxine; PM, pyridoxamine; PLP, pyridoxal 5′-phosphate; PNP, pyridoxine 5′-phosphate; PMP, pyridoxamine 5′-phosphate.

Pyridoxal 5′-phosphate (PLP) is the biologically active form of vitamin B_6_ and an essential cofactor in all domains of life. PLP-dependent enzymes catalyze diverse reactions, including transamination, elimination, decarboxylation, and racemization reactions (1–3). Organisms can obtain PLP by *de novo* synthesis and/or salvage of B_6_ vitamers from the environment. In general, bacteria and plants can synthesize PLP *de novo*, while higher eukaryotes, including humans, must salvage B_6_ vitamers from their environment. Two pathways have been described for *de novo* synthesis of PLP. The deoxyxylulose 5′-phosphate (DXP)-dependent pathway is found in Gammaproteobacteria including *Salmonella enterica*. This pathway combines glyceraldehyde 3-phosphate (G3P), pyruvate, and erythrose 4-phosphate through a series of steps that culminate in the formation of pyridoxine 5′-phosphate (PNP) (1). The final step in *de novo* synthesis of PLP from these precursors is catalyzed by pyridoxine 5′-phosphate/pyridoxamine 5′-phosphate oxidase (EC 1.4.3.5, PdxH), which forms PLP (Fig. 1). In the more common DXP-independent pathway, PLP is generated from G3P, glutamine, and ribose-5-phosphate through hydrolysis and condensation reactions catalyzed by a PLP synthase/glutaminase complex (EC 4.3.3.6, PdxS/PdxT in *Bacillus subtillus* and SNZp/SNOp in *Saccharomyces cerevisiae*).

Many organisms can satisfy a PLP requirement by salvaging one or more of the six B_6_ vitamers from the environment. Enzymes involved in salvage are broadly conserved across organisms and consist of the oxidase, PdxH, and one or more kinases (Fig. 1) (4, 5). The pathway used to salvage PLP in *S. enterica* includes kinases PdxK or PdxY (EC 2.7.1.35) (6, 7). Once phosphorylated, vitamers PNP and pyridoxamine 5′-phosphate (PMP) can be oxidized by PdxH to generate PLP. PLP can additionally be generated from PMP via transamination reactions that are independent of PdxH (8–10).

PLP contains a reactive aldehyde group that binds a conserved lysine in PLP-dependent enzymes through a Schiff base linkage and mediates diverse reaction mechanisms. The reactivity of the aldehyde has led to the prevailing assumption that levels of PLP are tightly controlled in the cell to prevent reactions that might inhibit enzymes and other cellular components (11–13). Proteins of the highly conserved, yet biochemically uncharacterized, PLP-binding protein family (COG0325) impact vitamin B_6_ homeostasis in eukaryotic and bacterial model systems (8, 14–16). Several names have been used for members of this family, including PROSC [human (17, 18)], YggS [*Escherichia coli*, *S. enterica* (8, 19)], YlmE [*Bacillus*, *Streptomyces* (20)], and PipY [*Cyanobacteria* (21)]. The convergence of data implicating these proteins in B_6_ homeostasis led to the designation PLPHP (PLP homeostasis protein), which provides a means to unify the literature across organisms (22). In humans, variants of PLPHP are biomarkers for B_6_-dependent epilepsy, which is characterized by disrupted B_6_ vitamer pools in cerebrospinal fluid and plasma (17, 18, 22, 23). The absence of functional PLPHP (designated YggS in *S. enterica*) results in disrupted B_6_ vitamer pools, including significantly elevated endogenous PNP pools in at least *S. enterica*, *E. coli*, and *S. cerevisiae* model systems (8, 24, 25). In *S. enterica* and *E. coli*, the absence of functional YggS (a PLPHP homolog) results in a significant accumulation of PLP in growth media (8).

Efforts to define the biochemical function of PLPHP have been complicated by the pleiotropic, and often indirect, effects associated with the loss of protein function. In this study, a mutation that prevents the exogenous accumulation of PLP from a *yggS* mutant in *S. enterica* was identified. Analysis of the suppressor mutation determined that when purines are limited, *yggS* mutants fail to accumulate PLP in growth media. Purine limitation did not affect additional consequences of a *yggS* mutation, thus isolating one phenotype caused by a *yggS* mutation and providing a means to better understand the one connection of YggS to cellular physiology in *S. enterica*.

## RESULTS AND DISCUSSION

**Fig 2:**
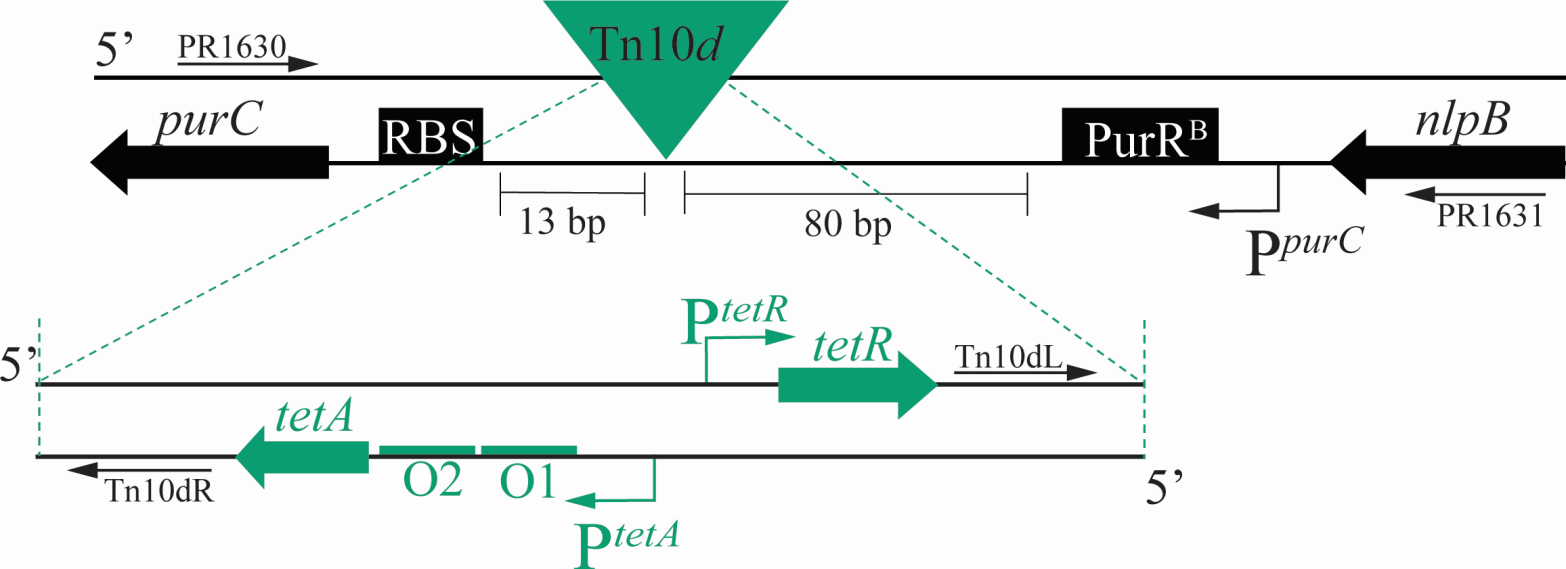
Position of the *purCp* insertion in the *S. enterica* genome. The intergenic region between the *purC* and *nlpB* genes in *S. enterica* is schematically shown and is not to scale. Gene locations are indicated with thick arrows in the orientation of their transcription. Elements of the native *purC* intergenic region are shown in black including ribosomal binding site (RBS) and the binding site of the PurR protein (PurR^B^). Elements of the inserted transposon (Tn*10*d) are shown in green. Transcription of *tetA* can read through to *purC* and is repressed by TetR unless tetracycline is present. Primer binding sites used in the analysis of genetic location are indicated. Initiation of transcription begins at the *purC* promotor (P*^purC^*) and is indicated by an arrow.

*S. enterica* mutants lacking *yggS* accumulate approximately eightfold more PLP in minimal glucose growth medium than an isogenic wild-type strain (8). A *yggS* mutant (DM15880) was subjected to transposon mutagenesis [Tn*10*(*d)Tc* (26)], and the resulting tetracycline-resistant clones were screened for decreased exogenous accumulation of B_6_ vitamers. The growth medium from mutants of interest was screened for accumulation of B_6_ vitamers by bioassay (Materials and Methods). Notably, the bioassay detects the presence of all B_6_ vitamers and is nonspecific with respect to the specific vitamer identity (i.e., PL, PM, PN, or phosphorylated derivatives). However, a previous study showed PLP was the primary B_6_ vitamer accumulating in growth medium of a *S. enterica yggS* mutant (8). From the initial screen, one clone that accumulated significantly less B_6_ vitamer in minimal growth medium than the parental *yggS* strain was identified. The putative mutant was used as donor to transduce the parental strain to tetracycline resistance (Tet^R^). Each of the Tet^R^ transductants tested (24) failed to accumulate B_6_ in their growth medium, showing the Tn*10(d*) insertion was responsible for the suppressor phenotype. The location of the Tn*10(d*) insertion was determined by PCR using arbitrary primers (27) and found to be inserted after nt*2,602,*853 in the *S. enterica* serovar Typhimurium strain LT2 genome (NCBI reference sequence: NC_003197.2). Thus, the insertion is in the intergenic region between the *purC* and *nlpB* genes in the genome. For simplicity, the mutation caused by this transposon is designated *purCp* throughout (Fig. 2). The location of the *purCp* insertion was confirmed by Sanger sequencing of PCR products generated from primers flanking the relevant region. The *purC* gene encodes 5′-phosphoribosyl-5-aminoimidazole-4-*N*-succinocarboxamide synthetase (EC 6.3.2.6), an enzyme that catalyzes the ligation of 5-amino-1-(5-phospho-D-ribosyl) imidazole-4-carboxylate (CAIR) and aspartate in the purine biosynthetic pathway. The *nlpB* gene encodes the BamC lipoprotein, a component of the BAM complex that is responsible for assembly and insertion of β-barrels into the outer membrane.

### Expression of *purC* is conditionally disrupted by the *purCp::Tn10(d)Tc* insertion

**Fig 3:**
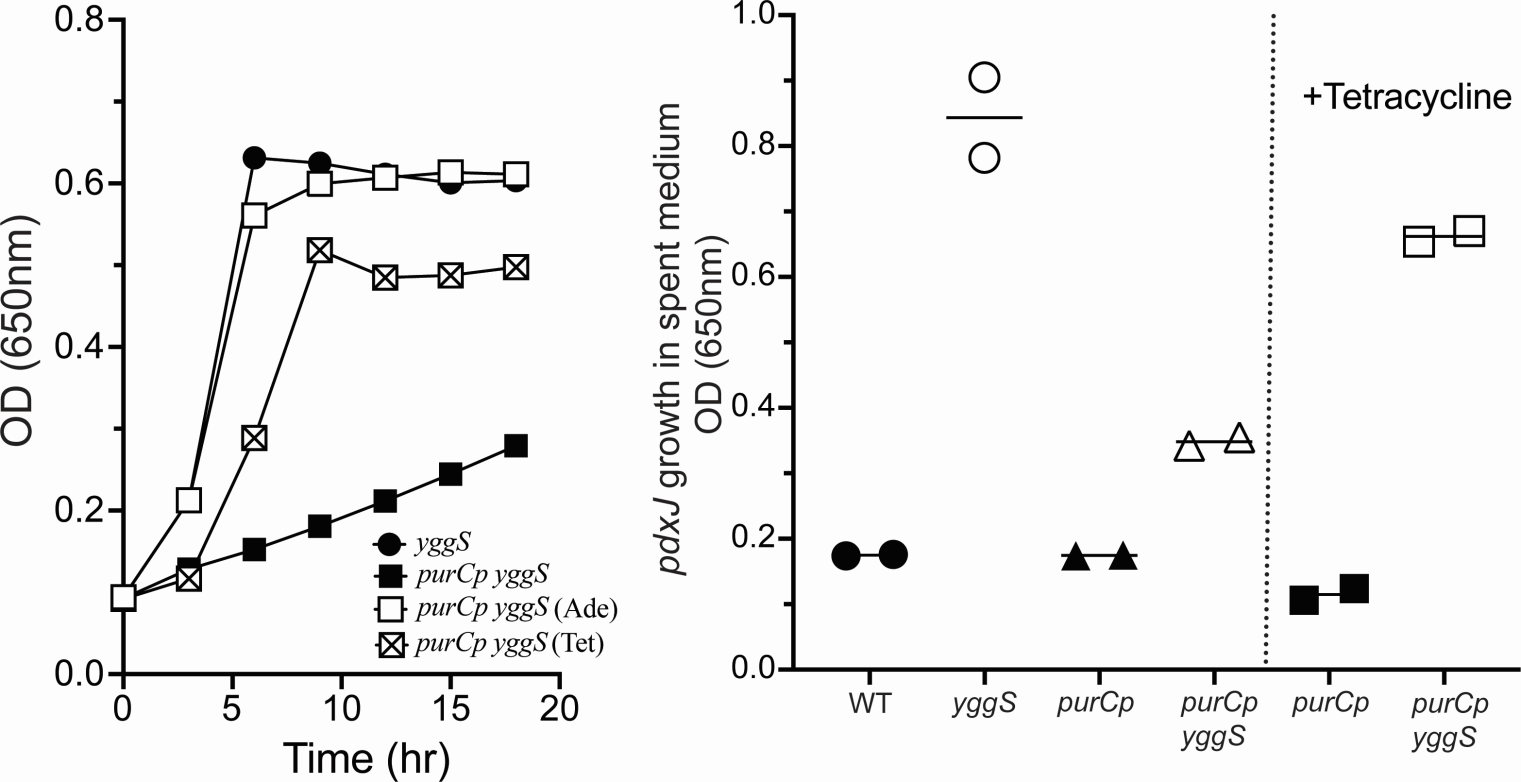
The *purCp* insertion suppresses a *yggS* mutant phenotype. Growth of *yggS* (circles) and *purCp yggS* (squares) mutant strains is shown in the left panel. Strains were grown in minimal glucose (solid symbols), with 150 µM adenine (open symbol) or 20 µg/mL tetracycline (hatched symbol) added. Growth was determined by monitoring optical density (OD) at 650 nm of three biological replicates over time. Error bars represent standard deviation from the mean and, when not visible, were covered by the symbol. Accumulation of extracellular B_6_ vitamers in spent medium was measured by bioassay and is shown in the right panel. The Y axis reflects the final OD_650_ reached by a *purJ* mutant (DM15964), which determines the presence of B_6_ vitamers in the medium (see Materials and Methods). The X axis depicts the source strain for the spent medium. Source strains were grown in minimal glucose medium or with tetracycline added where indicated. Significance was determined by paired two-tailed Student’s *t*-test and is denoted with an asterisk (*) when *P* < 0.05.

The *purCp* mutation caused a significant growth defect on minimal medium when it was present in either the wild-type (data not shown) or *yggS* mutant backgrounds (Fig. 3A). The map location, together with the growth data, suggested that the insertion disrupted transcription of *purC* and resulted in a partial limitation for purines. Supplementation with 150 µM adenine relieved the purine limitation and restored full growth (Fig. 3A). Significantly, the transposon encodes TetR, a repressor that regulates the internal promoters for *tetA* and *tetR*. In the presence of tetracycline, TetR repression is relieved and expression proceeds from these promoters (28). The location of the transposon insertion suggests that expression from the *tetA* promoter could read through and increase expression of *purC* (26), consistent with the increased growth allowed by tetracycline (20 µg/mL) (Fig. 3A). In the context of these growth data, B_6_ accumulation was quantified in relevant strains and growth media. Bioassays determined that the accumulation of B_6_ vitamers characteristic of a *yggS* mutant was eliminated by the presence of the *purCp* insertion (Fig. 3B). The accumulation of vitamers in the medium was restored when the *purCp yggS* strain (DM17738) was supplemented tetracycline (20 µg/mL). These data suggested a correlation between growth rate and B_6_ accumulation in the *yggS* mutant strain with the *purCp* mutation.

### Purine limitation reduces accumulation of B_6_ vitamer(s) in growth medium

**Fig 4:**
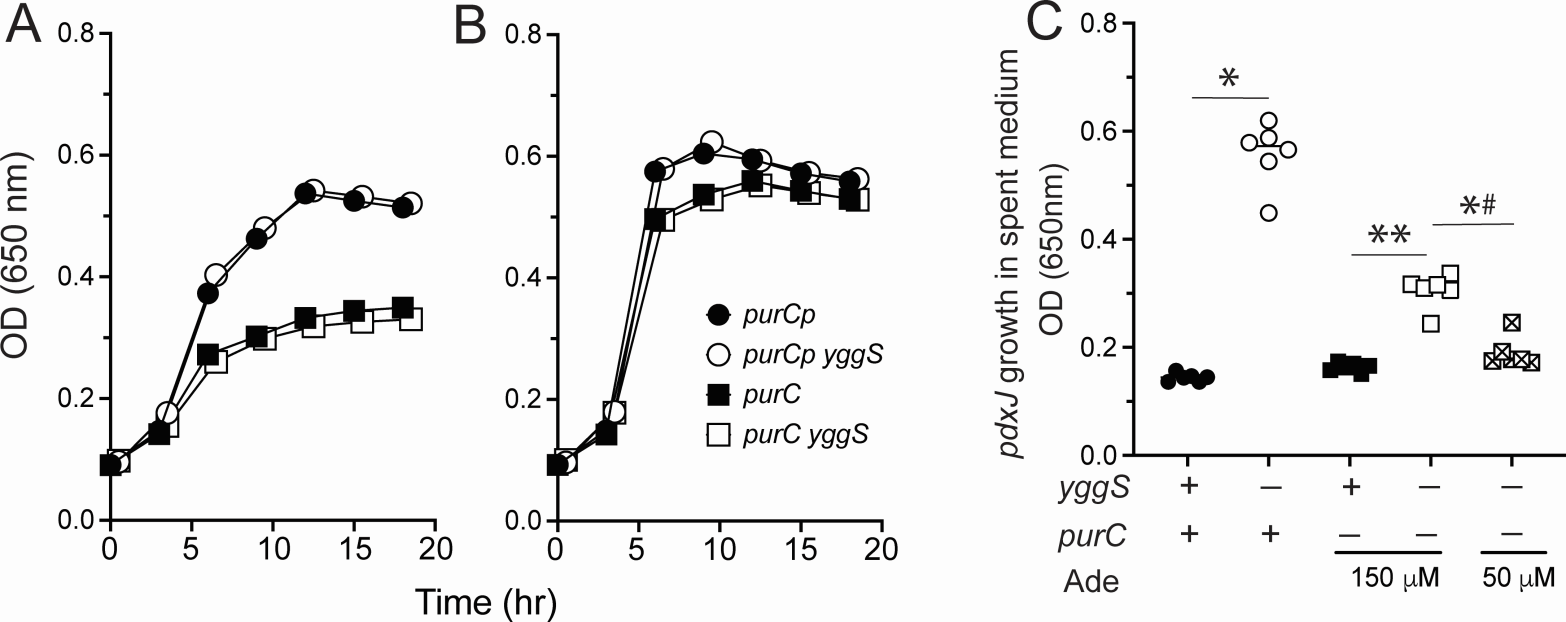
Analysis of adenine limitation strains lacking *purC*. (**A, B**) Growth of *purCp* mutant strain (closed circles), *purCp yggS* (open circles), Δ*purC* (closed squares), and Δ*purC yggS* (open squares) is shown. Strains were grown in minimal glucose supplemented with 50 µM (**A**) or 150 µM (**B**) adenine. Growth was monitored by OD at 650 nm of three biological replicates. Error bars represent standard deviation from the mean and are covered by the symbol when not visible. (**C**) Extracellular B_6_ accumulation was determined by bioassay, as described in Materials and Methods. The Y axis depicts the final OD_650_ reached by a *pdxJ* mutant (DM15964) grown in the spent media of six biological replicates. Spent media were generated from mutants grown in minimal medium or supplemented with adenine at the concentration indicated. The X axis depicts the relevant genotype (Δ*purC* or Δ*purC* yggS) of the source strain and the adenine (if any) present in the growth medium. Significance was determined by paired two-tailed Student’s *t*-test and is denoted with an asterisk (*) when *P* < 0.05. When relevant, significance was determined by paired two-tailed Student’s *t*-test with Bonferroni correction for multiple comparisons (*P* < 0.025) and is denoted with a double asterisk (**) while (*#) reflects *P* = 0.047 in the same test.

The results above suggested that the decrease in external B_6_ vitamer accumulation correlated with a purine limitation sensed by the cells. Strains with and without a *yggS* mutation and lacking all PurC activity due to an insertion mutation in the *purC* gene were constructed (Fig. 4). Spent medium from each of the strains grown in low (50 µM) and moderate (150 µM) adenine was monitored for B_6_ vitamer accumulation (Fig. 4C). The presence of 150 µM adenine increased the vitamer accumulation in the *yggS* derivative in comparison to the same strain grown with 50 µM adenine. Notably, vitamer accumulation was not restored to the level anticipated if adenine had fully eliminated the effect of the *purC* mutation. Growth data showed that strains lacking *purC* retained a slight growth defect in the presence of 150 µM adenine (Fig. 4B). Although 150 µM adenine restored full growth to *purCp*-containing strains, 500 µM adenine was required to restore full growth of the strains completely lacking PurC (data not shown). This difference was not unexpected since *purCp*-containing strains retain the ability to synthesize purines due to low expression of *purC*. In total, these data suggested that full supplementation of the purine requirement of a *purC yggS* mutant is needed to fully restore B_6_ vitamer accumulation in spent medium. In combination with the results from strains carrying *purCp*, these data confirm a correlation between purine limitation and extracellular accumulation of B_6_ vitamers from a *yggS* mutant.

**Fig 5:**
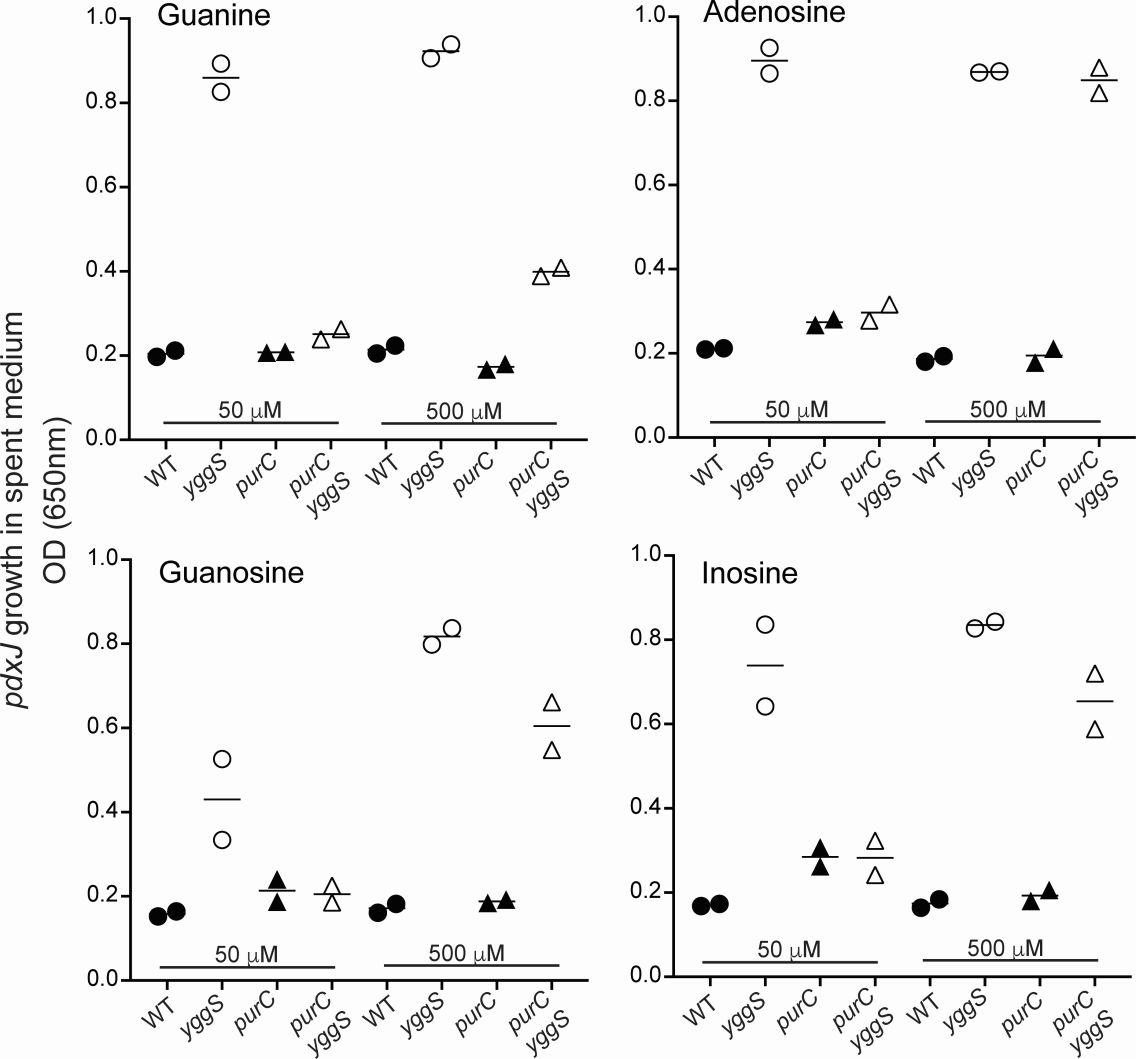
Supplementation with different purines restores exogenous B_6_ accumulation. Strains of the indicated genotype were grown with each of two concentrations of purines as noted. Spent media from two biological replicates were screened for accumulation of B_6_ vitamers by bioassay as described in the text. The Y axis reflects the presence of B_6_ vitamers in the medium as determined by the final OD_650_ reached after growth of a *pdxJ* mutant. The X axis shows the source strain for the spent medium.

The impact of purine limitation on B_6_ accumulation was not only relevant to adenine. When provided in excess, four non-adenine purine sources restored the accumulation of B_6_ in the growth medium of a *purC yggS* strain (Fig. 5). When supplemented with a limiting concentration (50 µM) of guanine, adenosine, guanosine, or inosine, the *purC yggS* double mutant accumulated levels of B_6_ vitamers in spent media that were indistinguishable from those of the *purC* single mutant strain. Accumulation of B_6_ vitamers was restored in a *purC yggS* strain when the purine sources were provided in excess (500 µM). The incomplete restoration with guanine was attributed to the fact that the phosphoribosyl transferase required for purine base salvage has a lower affinity for guanine than the other bases (29).

**Fig 6:**
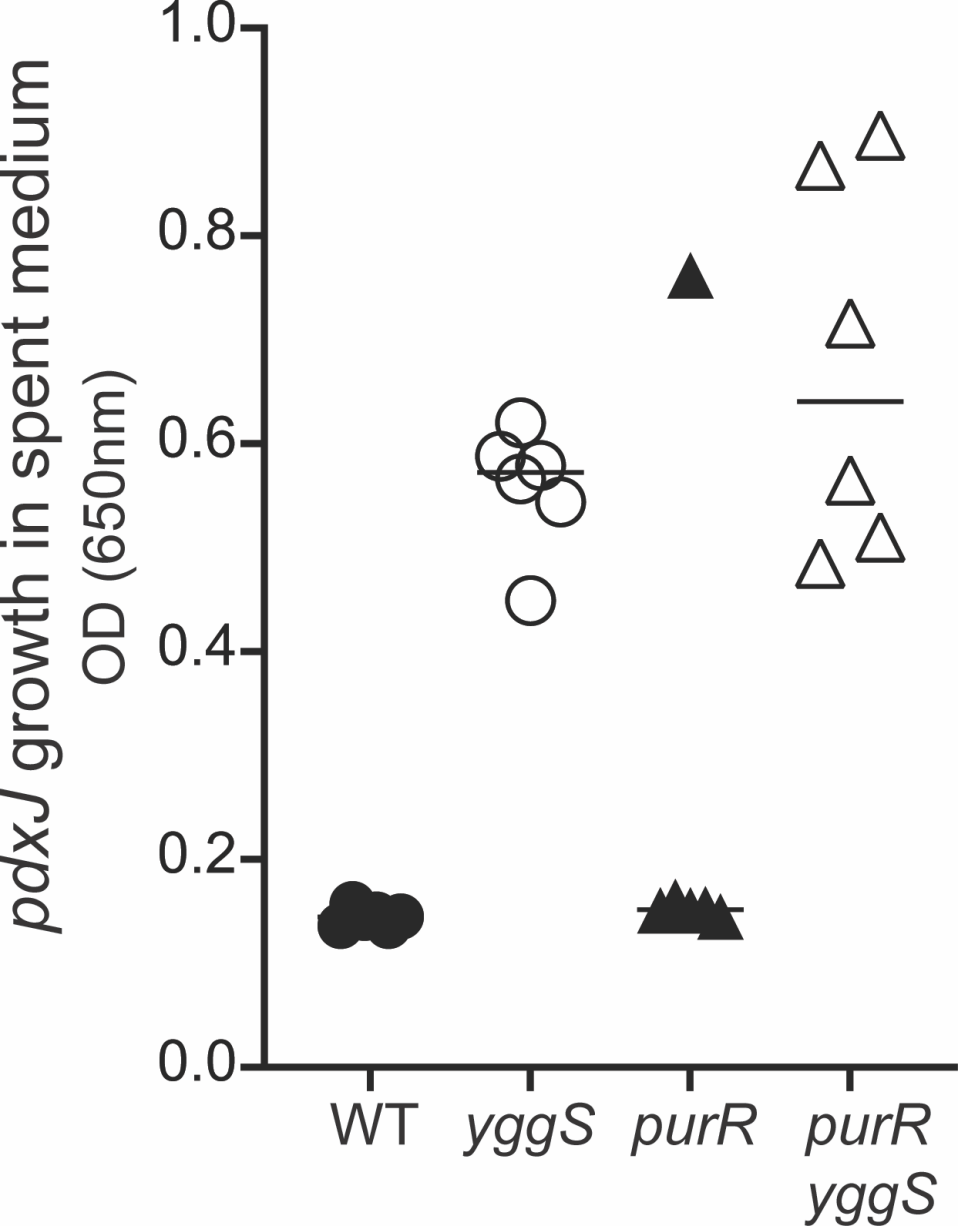
The PurR regulon is not the cause of PLP accumulation. Spent media of *purR* mutants grown in minimal glucose medium were screened for B_6_ accumulation by bioassay. The Y axis reflects the presence of B_6_ vitamers in the medium as determined by the final OD_650_ reached after growth of a *pdxJ* mutant. The X axis depicts the source strain for the spent medium. The *yggS* derivative of each pair accumulated significantly more B_6_ as determined by paired two-tailed Student’s *t*-test (*P* < 0.05).

PurR is a transcriptional repressor that binds DNA in the presence of purine effectors (30, 31). Thus, purine-limited growth induces the expression of genes in the PurR regulon (32). If the effect of purine limitation in a *yggS* mutant is mediated by the absence of PurR repression, a *purR* mutation would be expected to phenocopy the *purCp* mutation. A lesion in *purR* did not impact the accumulation of B_6_ vitamers in the spent medium of a *yggS* mutant (Fig. 6). These data eliminated a simple role for PurR in the accumulation of B_6_ vitamers from a *yggS* mutant.

### Purine limitation does not affect other *yggS* mutant phenotypes

**Fig 7:**
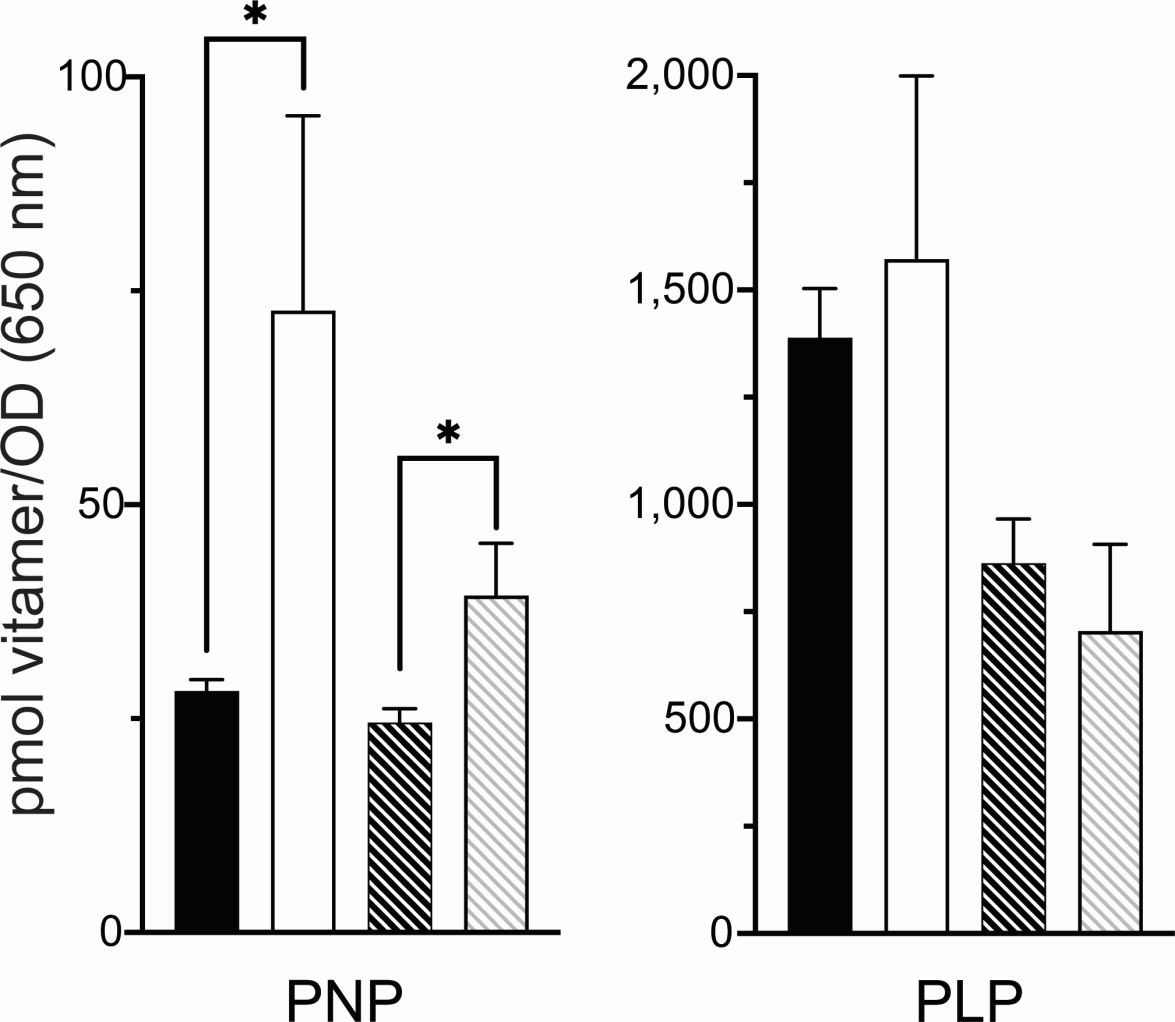
Intracellular PNP accumulation is not altered by purine limitation. Levels of intracellular phosphorylated B_6_ vitamer pools using three biological replicates were determined by HPLC. Data were normalized to cell OD_650_. Error bars represent standard deviation from the mean. Comparisons of each vitamer pools were made between Wildtype (black filled) and *yggS* (unfilled) strains or between *purCp* (black stripes) and *purCp yggS* (gray stripes) strains. Significance was determined by paired two-tailed Student’s *t*-test with Bonferroni correction for multiple comparisons (*P* < 0.0083) and is denoted with an asterisk (*).

In addition to exogenous B_6_ vitamer pools, the lack of *yggS* alters endogenous B_6_ vitamer pools. In particular, internal PNP levels are significantly elevated in a *yggS* mutant (8, 15). The effect of purine limitation caused by the *purCp* mutation on internal pools of PLP and PNP in a *yggS* mutant was determined (Fig. 7). The status of purine biosynthesis in the *yggS* mutant did not impact the ratio of internal pools of PNP or PLP vitamers. In addition to perturbing vitamer pools, a *yggS* mutation generates a synthetic phenotype in strains lacking aspartate aminotransaminase (EC 2.6.1.1, AspC). *S. enterica* strains lacking AspC can grow on minimal medium because the aromatic-amino-acid transaminase (EC 2.6.1.57, TyrB) has weak aspartate aminotransferase activity (33, 34). In contrast, an *aspC yggS* double mutant fails to grow on multiple carbon sources in the absence of added aspartate (35). An *aspC* mutant grows in minimal glucose media, while the *aspC yggS* mutant does not (reaching a final OD_650_ of 0.89 ± 0.02 vs 0.28 ± 0.17, respectively). The *purCp* mutation was transduced into both an *aspC* and an *yggS aspC* mutant background, and growth of the resulting strains was monitored. In minimal glucose media, the presence of the *purCp* mutation had no detectable effect on the growth of an *aspC* mutant or of the parental *aspC yggS* strain (final OD_650_ 0.85 ± 0.2 and 0.24 ± 0.04, respectively). In total, the above results indicated that purine limitation reversed one consequence of a *yggS* mutation but did not impact others.

### PLP synthesis is not affected by purine limitation

**Fig 8:**
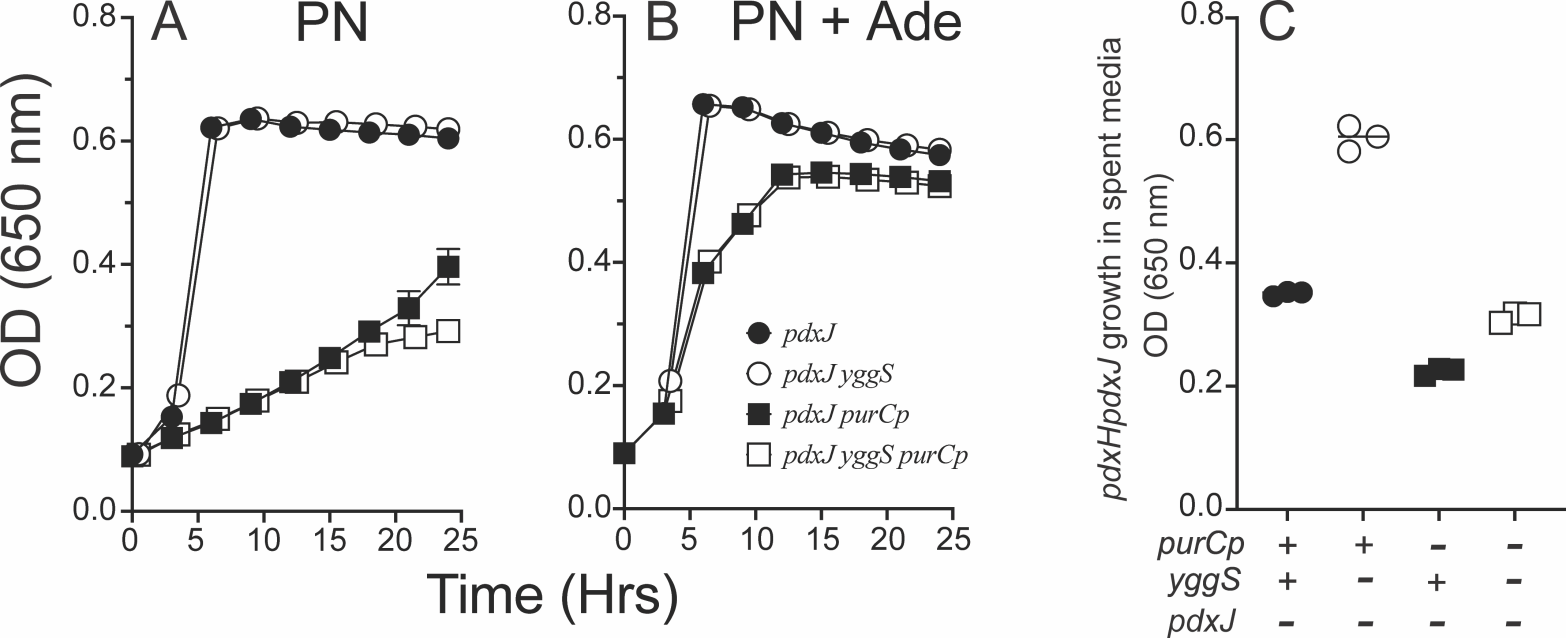
*De novo* PLP synthesis is not required for purine limitation to blunt B_6_ accumulation. Growth of *pdxJ* strains in minimal glucose containing 1 µM PN (**A**) and supplemented with 50 µM adenine (**B**) is shown above. Growth analysis of *pdxJ* (closed circles), *pdxJ purCp* (closed squares), and their *yggS^-^* derivatives (open shapes) was performed by monitoring OD at 650 nm of three biological replicates. Error bars represent standard deviation from the mean and are covered by the symbol if not visible. (**C**) Screening of extracellular B_6_ vitamer accumulation was performed in the spent media of three biological replicates. The bioassay measured the final OD_650_ of a PLP auxotroph that is specific for PL(P) and PM(P) vitamers (*ΔpdxJ pdxH*). Spent media were generated from the indicated strains grown in minimal glucose with 1 µM PN and 50 µM adenine. The *yggS* derivative of each pair accumulated significantly more B_6_ as determined by paired two-tailed Student’s *t*-test (*P* < 0.05).

One scenario to explain the effect of the *purCp* mutation suggested purine limitation could result in decreased PLP synthesis. In this scenario, the balance of endogenous vitamer pools would not be altered, but rather, the total amount of B_6_ vitamers would decrease, leaving a deficit of PLP that would otherwise accumulate exogenously. This possibility was addressed by blocking the *pdxJ* gene (encodes PLP synthase, EC 2.6.99.2) to prevent *de novo* PLP synthesis. With PN provided as the sole source of PLP, an impact of the *purCp* insertion on PLP synthesis would be eliminated. Four relevant strains were constructed and assessed for their growth and accumulation of B_6_ vitamers exogenously (Fig. 8). Strains were grown with PN (1 µM) and with or without a limiting level of adenine (50 µM). As expected, growth of the *pdxJ purCp* strain was less than the wild type with 50 µM adenine provided. The bioassay for B_6_ accumulation utilized a *pdxJ pdxH* strain (DM16397) to allow discrimination between B_6_ vitamers. Since PN was added to the growth medium, an indicator strain that only utilized PL(P) and PM(P) vitamers was necessary. The absence of *de novo* PLP synthesis did not affect the accumulation of B_6_ vitamers in the growth medium of a *yggS* mutant. Despite the lack of *de novo* PLP synthesis, B_6_ accumulation was still minimized by the *purCp* mutation when growth medium was not fully supplemented for purines. These data supported the conclusion that purine limitation was not reducing flux through the *de novo* biosynthetic pathway for PLP as a mechanism of decreasing its external accumulation.

### ATP limitation does not impact B_6_ accumulation from a *yggS* mutant

**Fig 9:**
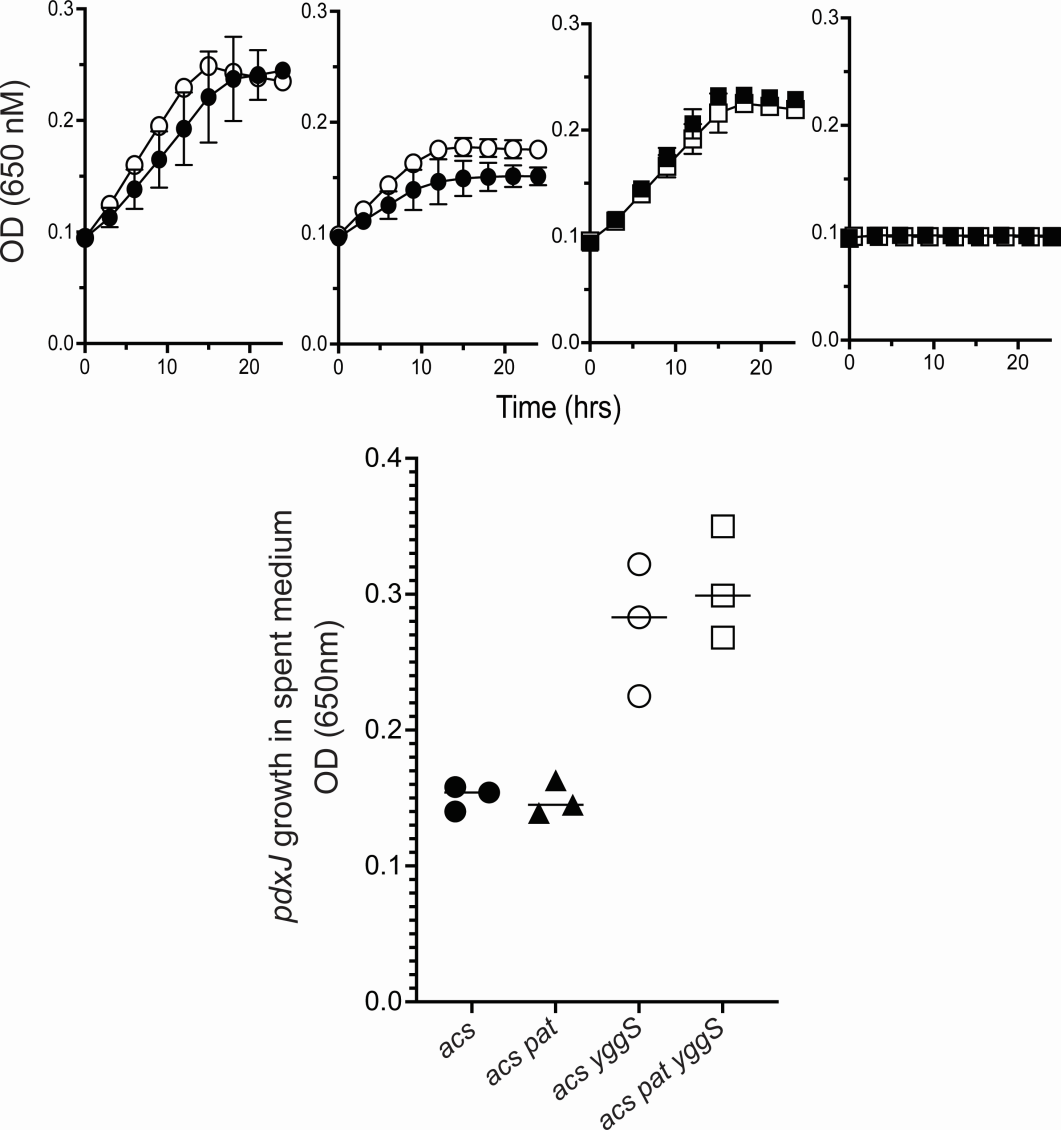
ATP limitation does not prevent exogenous accumulation of B_6_. Strains were grown in 10 mM acetate minimal media with 50 µM methionine. (**A–D**) Growth of *acs* (closed circle), *acs yggS* (open circle), *acs pat* (closed square), or *acs pat yggS* (open square) carrying *acs* on a plasmid is shown above. Strains were uninduced (**A and C**) or induced (**B and D**) with 0.2% arabinose. Growth analysis was performed by monitoring OD at 650 nm of three biological replicates. Error bars represent standard deviation from the mean. (**E**) All strains carried *acs* on a plasmid and were induced with 0.2% arabinose for 4.5 hours. Screening of spent media was performed using a bioassay measuring the final OD_650_ of a *pdxJ* mutant (DM16461) in the spent media of three biological replicates.

Additional scenarios were considered to explain the effect of purine limitation on exogenous vitamer accumulation from a *yggS* mutant. Cells grown in purine-limited conditions have lowered ATP levels (36), which might impact vitamer export. Strains previously characterized in *S. enterica* were used to address the possibility that exogenous accumulation of B_6_ vitamers in a *yggS* mutant was prevented by a decrease in energy charge in the cells. Briefly, in *S. enterica*, Acs (acetyl-coenzyme A synthetase, EC 6.2.1.1) is required for growth on 10 mM acetate, where it generates acetyl-CoA in a two-reaction process. The first reaction consumes ATP and acetate to generate acetyl-AMP, while the second reaction combines acetyl-AMP with CoA to generate acetyl-CoA. Critically, if Acs is overexpressed *in trans* and acetylation of Acs by Pat (an N-acetyltransferase encoded by *pat*) is prevented, ATP pools are depleted, which results in a growth defect (37). Introduction of a *yggS* mutation into the relevant strains did not alter their growth indicating this lesion did not prevent depletion of the ATP pool (Fig. 9). Thus, as expected, *acs* and *acs yggS* mutant strains grew in 10-mM acetate minimal medium with or without induction of Acs *in trans*, while the *acs pat* and *acs pat yggS* mutant strains did not grow when Acs was overexpressed.

Spent media of the four strains overexpressing Acs were screened for extracellular B_6_ vitamer accumulation. Because *pat* mutant strains are unable to grow when Acs is overexpressed, media tested for vitamer accumulation were obtained from resting cells. Strains were grown to stationary phase without arabinose, pelleted, and resuspended in 10 mM acetate medium with arabinose (to induce *acs*). As anticipated, no B_6_ vitamers were detected in media from the two strains with functional YggS protein. The *yggS* mutant strains accumulated similar levels of B_6_ vitamers, though less in total than in other experimental conditions. The reduced accumulation can be explained by the use of resting cells, a conclusion supported by the increase in accumulation in the medium over time (data not shown). Significantly, the increase in B_6_ accumulation caused by a *yggS* mutation occurred when *acs* was overexpressed. There was no significant difference in this accumulation with or without the presence of a *pat* mutation, which would result in decreased energy charge in the cells, as determined by a paired two-tailed Student’s *t*-test (Fig. 9). These data supported the conclusion that ATP limitation, generated by the *purCp* insertion, was not the mechanism by which B_6_ vitamer accumulation was altered by this mutation.

### Growth rate does not affect B_6_ vitamer accumulation from a *yggS* mutant

**Fig 10:**
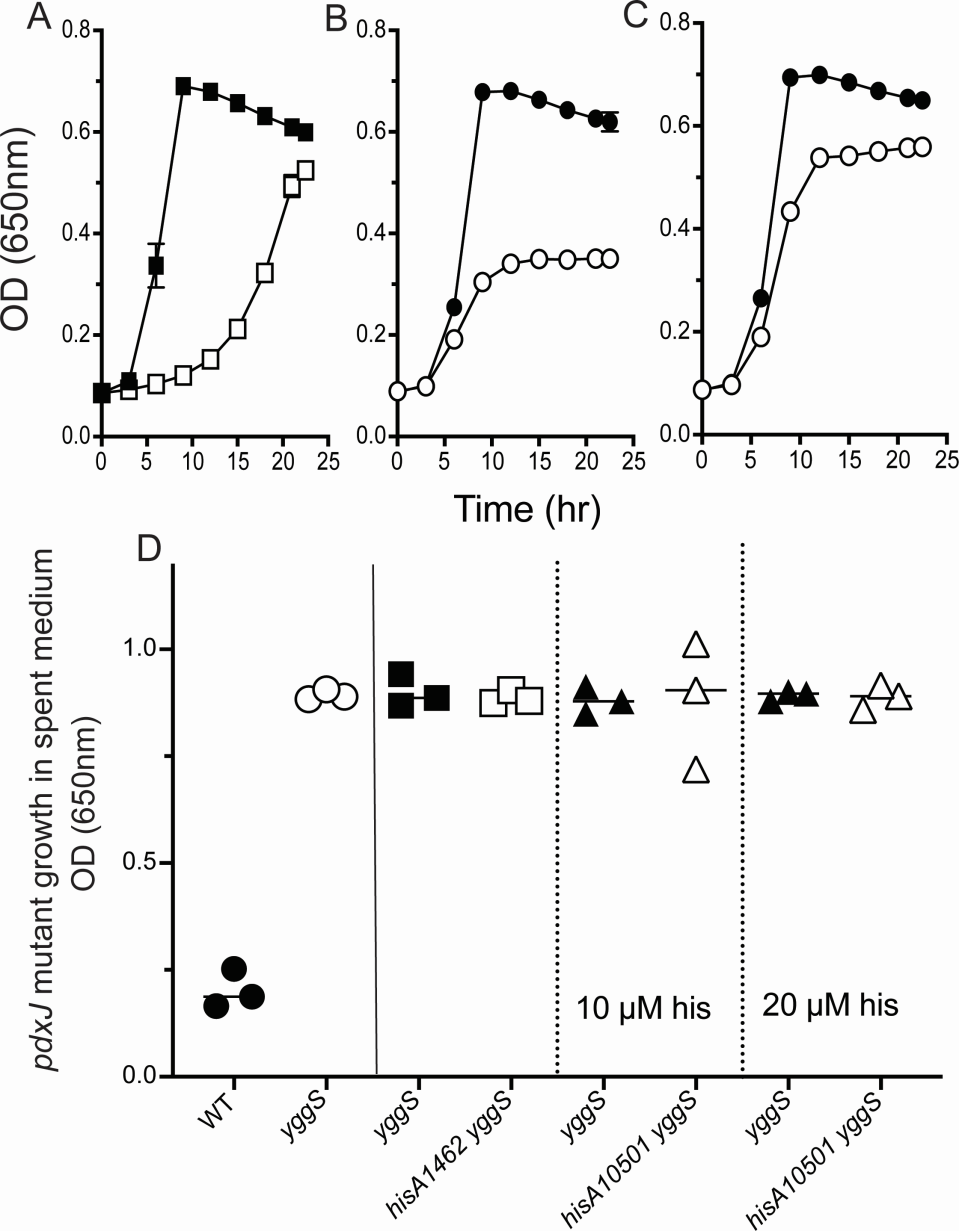
Decreased growth rate fails to prevent B_6_ exogenous accumulation. Isogenic *hisA1462 gnd yggS* (open squares) and *gnd yggS* (closed squares) stains were grown in minimal glucose medium (**A**). Isogenic *hisA10501 gnd yggS* (open circles) and *gnd yggS* (closed circles) strains were grown in minimal glucose supplemented with either 10 µM (**B**) or 20 µM (**C**) histidine. Growth analysis was performed by monitoring OD at 650 nm of three biological replicates. Error bars represent standard deviation from the mean and, where not visible, were covered by the symbols. (**D**) Screening of spent media was performed by a bioassay, which measured the final OD_650_ of a PLP auxotroph (DM16461) reached in the spent media. Spent media were generated by growing three replicates of the indicated strains in minimal glucose media with or without histidine as indicated.

Strains limited for purines have a reduced growth rate. Metabolic differences caused by changes in growth rate could potentially impact B_6_ accumulation from a *yggS* mutant. Two mutant alleles of *hisA* that compromise *de novo* synthesis of histidine addressed a role for growth rate in determining B_6_ vitamer accumulation. *hisA10501* and *hisA1461* encode variants of 1-(5-phosphoribosyl)-5-(38) imidazole-4-carboxamide (ProFAR) isomerase (EC 5.3.1.16). Both variants are recessive and encode HisA enzymes with reduced ProFAR isomerase activity (38). Stains were constructed to determine the impact of the *hisA* alleles on the vitamer accumulation phenotype caused by a *yggS* mutation. As anticipated, slowed growth was observed in strains containing the *hisA1461* allele. The *hisA1461* mutation had no impact the vitamer accumulation of a *yggS* mutant strain (Fig. 10). The *hisA1501* allele prevented growth in minimal medium, and 10 or 20 µM histidine supplemented the medium to allowed growth to different levels. Significantly, neither media condition impacted B_6_ accumulation from the *yggS* mutant strains. These data supported the conclusion that growth rate does not affect B_6_ accumulation.

### Conclusions

Strains lacking the *yggS* gene accumulate PLP in the growth medium. An insertion described herein results in decreased expression of *purC* and prevents accumulation of the B_6_ vitamers in the medium. The relevant *purCp* insertion does not affect other phenotypes described for a *yggS* mutant, indicating purine limitation specifically impacts the accumulation of B_6_ vitamers. Consequences of purine limitation include (i) derepression of the PurR regulon, (ii) limitation of ATP, possibly affecting the energy charge of the cell, and (iii) slowed growth rate. When probed, none of these consequences recapitulated the effect of limiting purines in a *yggS* mutant strain.

A characterized mechanism for exporting metabolites from bacterial cells involves multiple drug efflux pumps (39, 40). It is possible that one or more of these pumps is involved in generating the exogenous PLP accumulation caused by a *yggS* mutant. Exploring a connection between efflux pumps and vitamer accumulation in *yggS* mutants could further our understanding of how these strains export PLP. It remains unclear not only how but why a strain would release such a valuable metabolic cofactor to the medium. Further studies on the function of YggS may provide hints to these biological questions in addition to a better understanding of the physiological role of YggS and its conserved homologs.

## MATERIALS AND METHODS

### Media and chemicals

Rich medium was Difco Nutrient Broth (NB, 8 g/L) with NaCl (5 g/L). Minimal medium was no-carbon E salts (41) supplemented with trace elements (42) and MgSO_4_ (100 µM). Glucose (11 mM) was added as the sole carbon source unless otherwise stated. Nutritional supplements and their concentrations are noted in the text. Antibiotics were used as needed and included tetracycline (20 µg/mL), kanamycin (100 µg/mL), and ampicillin (150 µg/mL). All chemicals were purchased from Millipore-Sigma.

### Bacterial strains, plasmids, primers, and growth quantification

All strains used in this study are derivatives of *Salmonella enterica* serovar Typhimurium strain LT2 (*S. enterica*) or *E. coli* MG1655 and are listed with their source in Table 1. When needed, strains were constructed by transduction with phage P22 HT105/1 *int-201* selecting a suitable marker (43). Transductants were made phage free as described (44). Plasmids and primers are described in Table 2.

**TABLE 1:** Bacterial strains

Strain	Relevant genotype^*a*^	Source
*S. enterica* strains		
TT10424	*proAB47* [*F′ pro+ lac∷* Tn*10d*(Tc)*^b^*]	JR Roth Laboratory
DM15847	Wild type	Laboratory collection
DM15880	*yggS644*::Cm *ΔaraCBAD*	Laboratory collection
DM15902	*yggS644*::Cm	Laboratory collection
DM15903	*yggS648*::Km	Laboratory collection
DM15948	*ΔyggS651*	Laboratory collection
DM15964	*ΔpdxJ664*	Laboratory collection
DM17667	*pdxJ662*::Km *ridA1*::Tn*10d*(Tc)	Laboratory collection
DM16397	*ΔpdxH673 ΔpdxJ664*	Laboratory collection
DM16461	*pdxJ666*∷Cm (*pBAD322G*)	Laboratory collection
DM17737	*purCp*::Tn*10d*(Tc) (*AKA purCp*)	This study
DM17738	*purCp*∷Tn*10d*(Tc) *yggS648::*Km	This study
DM17752	*purC2156*::MudJ^*c*^	This study
DM17799	*purC2156*::MudJ *yggS644*::Cm	This study
DM17772	*purR3150*::Kan4	This study
DM17798	*purR3150*::Kan *yggS644*::Cm	This study
DM18331	*gnd174*::MudJ *yggS644*::Cm	This study
DM18332	*gnd174*::MudJ *hisA1451 yggS644*::Cm	This study
DM18334	*gnd174*::MudJ *yggS644*::Cm	This study
DM18333	*gnd174*::MudJ *hisA10501 yggS644*::Cm	This study
JE9912	*Δacs2 (pBAD30.acs*)	(37)
JE9960	*Δacs2 pat10*∷MudJ (*pBAD30.acs*)	(37)
DM18265	*Δacs2 yggS644*∷Cm (*pBAD30.acs*)	This study
DM18267	*Δacs2 pat10*∷MudJ *yggS644*∷Cm (*pBAD30.acs)^d^*	This study
*E. coli* strains		
DM16876	BW25113 Δ*pdxJ*::Km	Laboratory collection
DM17365	MG1655 *ΔpdxJ::*Km (pCVI.*phoN*)	Laboratory collection

^*a*^ All mutations designated with two letter antibiotic resistance were developed by the Wanner method (45).

^*b*^ Tn*10d*(Tc) denotes defective transposon Tn10d16d17d (26).

^*c*^ MudJ is an abbreviation of MudI1734(lacZ+ kan+) (46).

^*d*^ Strain from the single-gene deletion collection in *Salmonella enterica sv Typhimurium 14028*s (47) was used as donor in transduction to construct strains.

**TABLE 2:** Plasmids and primers

Plasmid name	Plasmid insert	Backbone	Resistance
pNK972	*Tn10*-specific transposase	F′	Ampicillin
pCVI.*phoN*	*phoN*	pCVI (48)	Ampicillin
pBAD322G	none	pBAD322G (49)	Gentamycin
pBAD30.*acs*	*acs*	pBAD30 (50)	Ampicillin

Primer	Sequence (5′ to 3′)		
Arb1	GGCCACGCGTCGACTAGTACNNNNNNNNNNGATAT		
Arb6	GGCCACGCGTCGACTAGTACNNNNNNNNNNACGCC		
Tn10L	TCCATTGCTGTTGACAAAGGGAAT		
Tn10R	ACCTTTGGTCACCAACGCTTTTCC		
PR1630	CGCTTTACCACGATACAACT		
PR1631	CGGAGACTATAAATTGCAGGTTG		

Growth of *S. enterica* strains was determined quantitatively in liquid medium. Strains were grown overnight in 2 mL NB before subculturing (2.5% inoculum) into indicated medium (200 µL total volume). Where noted in the text, cells in the overnight culture were pelleted at 3,200 × *g* for 15 minutes and resuspended in an equal volume of 0.85% saline prior to inoculation. Growth was measured via optical density at 650 nm (OD_650_) in a 96-well plate using a BioTek ELx808 plate reader (BioTek Instruments, Winooski, VT). All graphs were created using Prism 9.5.1 (GraphPad Software, La Jolla, CA). Typically, three biological replicates were used and growth was plotted with error bars.

### Tn10*d* mutagenesis

Strain DM15880 (*yggS644::Cm*) strain containing pNK972 (51) was transduced to tetracycline resistance with a P22 lysate grown on strain TT10424. Transduction plates (NB, Tet) were printed directly to rich media plates containing EGTA (10 mM) and tetracycline and minimal glucose plates seeded with DM17365 (*E. coli ΔpdxJ::Km*/ pCVI.*phoN*). Strain DM17365 requires a source of B_6_ for growth and expresses PhoN, which is needed to salvage PLP (52). After replica printing to the seeded plates, a halo of growth is seen around transductant colonies that release enough B_6_ to support growth of the *E. coli* strain. *E. coli* was used as the seeded strain to avoid lysis by residual P22 present on the transduction plates.

Transductants that did not have a visible halo of growth around them were pursued as mutants of interest. Putative suppressors were screened with a bioassay (see below) for accumulation of B_6_ vitamer(s) in liquid medium. The transposon in each relevant mutant was moved into a naïve parental genetic background by transduction, and the resulting strain was confirmed phenotypically. If found to be causative, the transposon insertion was mapped using PCR with degenerate primers as described (27, 53). Sanger sequencing was outsourced to Eurofins, Louisville, KY. Sequence alignment to the *S. enterica* genome was performed using Geneious Prime 2022.1.1 (Biomatters Inc., Boston, MA). The location of the transposon insertion was confirmed by Sanger sequencing of PCR products generated using primers specific to the region.

### Bioassay for B_6_ vitamers

The bioassay for B_6_ vitamers in growth medium has been described (8). Cultures grown overnight in rich media were subcultured (120 µL) into minimal glucose medium (3 mL), with supplements as indicated. Cultures were grown to an OD_650_ of ~0.5 or as indicated in the text, measured by a GENSYS 30 Visible Spectrophotometer. Cells were pelleted at 3,200 × *g* for 15 minutes, and spent medium was filter sterilized and stored at −20°C. When relevant, 5 µg/mL gentamycin was added to the spent medium in place of filter sterilization. All spent medium was supplemented with trace elements, MgSO_4_ (100 µM) and glucose (11 mM), prior to use for growth of indicator *pdx* mutant strains. In general, a Δ*pdxJ* mutant (DM15964) was used as the indicator strain. When gentamycin was used in the spent media, the *pdxJ* mutant carried a vector encoding gentamycin resistance (DM16461). When tetracycline was present in the spent media, a *pdxJ* mutant resistance to tetracycline (DM17667) was used. When the PN vitamer was added to the growth medium, a *pdxJ pdxH* double mutant (DM16397) was used as indicator strain. The presence of B_6_ vitamer(s) in the spent media was reflected by growth of the appropriate strain as indicated by OD (650 nm). Growth was monitored in 96-well plates.

#### ATP limiting conditions

A modified bioassay was used when strains carrying a deletion in *acs* were involved. In this case, cells from overnight NB cultures (2 mL) were pelleted at 3,200 × *g* for 15 minutes and resuspended in minimal acetate (10 mM) medium (3 mL) supplemented with 50 µM methionine and 0.2% arabinose. Samples were taken after 1.5 and 3 hours of incubation at 37°C with shaking. In each sample, cells were pelleted at 3,200 × *g* for 15 minutes. Spent medium was supplemented with 5 µg/mL gentamycin, trace elements, MgSO_4_ (100 µM), and glucose (11 mM). The presence of B_6_ vitamer(s) in the sample was determined by growth of a gentamycin-resistant *pdxJ* mutant (DM16461). Growth was monitored in 96-well plates.

### Analysis of B_6_ vitamer content by high-performance liquid chromatography

High-performance liquid chromatography (HPLC) analysis of B_6_ vitamers has been described (8, 24). Briefly, strains were grown in minimal glucose media and harvested at an OD_650_ between 0.5 and 0.6. Cells were acquired by pelleting 3 mL of culture and removing the spent medium. Intracellular vitamers were extracted by treating cell pellets with five volumes (wt/vol) of 7% HClO_4_, incubating on ice for 15 minutes, and adding 2.5 volumes of 0.7 M K_2_CO_3_, before a final placement on ice for 15 minutes. Extraction mixtures were centrifuged at 17,000 × *g* for 15 minutes at 4°C, and the supernatant was used for analysis. Intracellular extraction mixtures were typically diluted threefold with ddH_2_O prior to injection on the HPLC.

Vitamer analysis used a Shimadzu Prominence HPLC System (Shimadzu Scientific Instruments Inc., Columbia MD). Extracts prepared as described above were injected (25 µL) into a 250 by 4.6 mm Cosmosil AR-II octyldecyl silane (ODS) column (5 m particle size; Nacalai USA Inc., San Diego, CA). Mobile phase A contained phosphoric acid (33 mM) and 1-octanesulfonic acid (8 mM) adjusted to pH 2.2, while mobile phase B contained 80% (vol/vol) acetonitrile. Vitamer pools were separated at 27°C using a linear gradient program as follows: 0% B to 1% B for 5 minutes, 1% B to 19% B for 5 minutes, 19% B to 28% B for 10 minutes, 28% B to 63% B for 5 minutes, 63% B for 5 minutes, 63% to 0% B for 5 minutes, and column equilibration with 100% A for 15 minutes. The total flow rate was 0.8 mL/minutes, and running time was 50 minutes. Online post-column fluorescent derivatization was used to detect B_6_ vitamers after column separation. Column elutant was mixed in-line with a 1-M potassium phosphate derivatization buffer (pH 7.5) containing sodium bisulfite (1 g/L) to increase fluorescence prior to detection (excitation at 328 nm and emission at 393 nm). Derivatization buffer was pumped by an ÄKTAdesign Pump P-920 (Amersham Pharmacia Biotech, Sweden) at a flow rate of 0.3 mL/minutes.
